# Enhancing Antibodies’ Binding Capacity through Oriented Functionalization of Plasmonic Surfaces

**DOI:** 10.3390/nano11102620

**Published:** 2021-10-05

**Authors:** Maria Laura Coluccio, Fabiana Grillo, Valentina Onesto, Virginia Garo, Cinzia Scala, Paola Cuzzola, Michela Calfa, Patrizio Candeloro, Francesco Gentile, Sergey Piletsky, Natalia Malara

**Affiliations:** 1 BioNEM Laboratory and Nanotechnology Research Center, Department of Experimental and Clinical Medicine, University “Magna Graecia” of Catanzaro, 88100 Catanzaro, Italy; coluccio@unicz.it (M.L.C.); paola.cuzzola@studenti.unicz.it (P.C.); michela.calfa@studenti.unicz.it (M.C.); patrizio.candeloro@unicz.it (P.C.); francesco.gentile@unicz.it (F.G.); 2 Department of Chemistry, University of Leicester, Leicester LE1 7RH, UK; fg90@leicester.ac.uk (F.G.); sp523@leicester.ac.uk (S.P.); 3 Institute of Nanotechnology, National Research Council (CNR-NANOTEC), Campus Ecotekne, via Monteroni, 73100 Lecce, Italy; valentina.onesto@nanotec.cnr.it; 4 Department of Health Science, University “Magna Graecia” of Catanzaro, 88100 Catanzaro, Italy; virginia.garo@studenti.unicz.it (V.G.); cinzia.scala@studenti.unicz.it (C.S.)

**Keywords:** IgG, metal enhanced fluorescence, plasmonic surface, surface functionalization, biosensor

## Abstract

Protein A has long been used in different research fields due to its ability to specifically recognize immunoglobulins (Ig). The protein derived from Staphylococcus aureus binds Ig through the Fc region of the antibody, showing its strongest binding in immunoglobulin G (IgG), making it the most used protein in its purification and detection. The research presented here integrates, for the first time, protein A to a silicon surface patterned with gold nanoparticles for the oriented binding of IgG. The signal detection is conveyed through a metal enhanced fluorescence (MEF) system. Orienting immunoglobulins allows the exposition of the fragment antigen-binding (Fab) region for the binding to its antigen, substantially increasing the binding capacity per antibody immobilized. Antibodies orientation is of crucial importance in many diagnostics devices, particularly when either component is in limited quantities.

## 1. Introduction

In the past 20 years the interest in combining bio recognition elements to sensors platforms and assays has risen exponentially [1]. The need for an accurate and specific recognition of target molecules in complex matrixes has led to a significant demand growth in the development of accurate detection platforms. 

Antibodies represent, even at the present day, the most used affinity reagents for a variety of different applications [2]. With a global research market size estimated at 3.4 billion in 2019 and a forecast revenue in 2027 of USD 5.6 billion [3] they have effectively revolutionized the area of therapeutics, diagnostics, separation, and purification science [4].

**Figure 1:**
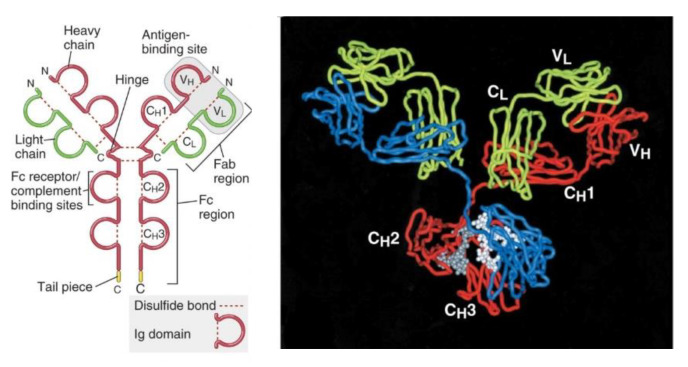
Schematic diagram of a secreted IgG molecule. The antigen-binding sites are formed by the juxtaposition of V L and V H domains. The heavy chain C regions end in tail pieces. The locations of complement- and Fc receptor–binding sites within the heavy chain regions. Reprinted with permission from Ref. [5]. Copyright 2017 Elsevier.

Antibodies structurally are molecules with a symmetric core composed of two identical light chains and two identical heavy chains [5]. Both the light chains and heavy chains contain a series of repeating homologous structural units that fold independently in a globular motif that is called an Ig domain. These macromolecular proteins of 150 kDa have two fragment antigen binding (Fab) domains where their cognate antigens bind with high selectivity and specificity. In addition, Igs display two fragment crystallizable (Fc) regions (Figure 1) that are the primary recognition site for e.g., cell surface receptors of effector cells, immune proteins, and other antibodies. These molecules can be divided into distinct classes and subclasses on the basis of differences in the structure of their heavy chain C regions. The classes of antibody molecules are also called isotypes and are named IgA, IgD, IgE, IgG, and IgM. In humans, IgA and IgG isotypes can be further subdivided into closely related subclasses, or subtypes, called IgA1 and IgA2 and IgG1, IgG2, IgG3, and IgG4. Among the five different classes of antibodies, IgGs are the most abundant Ab (human concentration 13.5 mg/mL) [5] and are also the second longest circulating protein in the blood stream [6]. 

IgGs have been applied in the therapeutics industry for many diseases, including autoimmune diseases, cancer, septicemia, and to neutralize various viral infections [7]. At the time of this article, 100 monoclonal antibody products had been approved by the FDA for therapeutical use [8].

Over the years ELISA has significantly improved in its specificity and sensitivity, reaching pM concentrations [9,10], and since their first diagnostic application in 1971, the range of platforms in which they are used has largely expanded. Antibody applications range from attaching them to fluorescent [11,12], gold, or magnetic beads [13,14,15,16,17], to many activated surfaces, gold, glass, carbon [18,19,20,21].

For most of these applications, the receptor antibody must be immobilized to a solid support. Over the years, many strategies have been explored, the most representative of each class are briefly highlighted in the following section.

**Figure 2:**
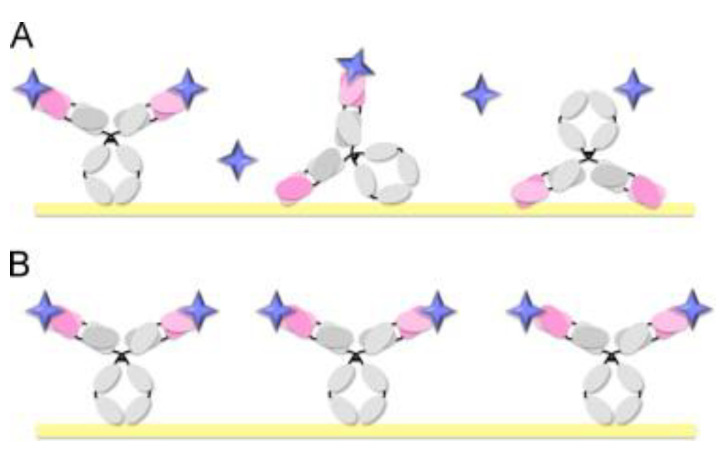
Two main approaches to antibody immobilization. (**A**) Random, and (**B**) site-directed antibody immobilization. Reprinted with permission from Ref. [27]. Copyright 2013 Elsevier.

The easiest way to immobilize antibodies is by passive adsorption, where no previous modification of either the antibody or the surface is needed, however, due to the non-covalent immobilization, relying on hydrophobic, van der Waals, and pi-pi interactions, antibodies can easily leach out during the washing steps effectively reducing the amount of antibody available for the binding [22]. In addition, a precise control over the orientation of antibodies is hardly achievable (Figure 2).

A more robust strategy is to covalently immobilize the antibodies on a plate using free amino and carboxyl groups naturally present in the antibody, and apply a standard EDC/NHS chemistry. This approach solves the issue relative to the antibody leach, however, due to the equal distribution of those groups on the whole protein, the problem relative to the random orientation still remains [4].

Many other strategies have been tried such as tagging the Fc region of the antibody with biotin and immobilizing on the plate streptavidin, taking advantage of the strongest known binding interaction in nature (biotin-streptavidin binding affinity k_d_ = ~fM) [23,24,25], or utilizing a well-known polyhistidine tag (His-tag), having affinity for metal ions (Ni^2+^, Co^2+^, Cu^2+^) [26]. Although the methods described will all orient the immobilization of IgG, they also present known problems as the necessity of labelling antibodies and the relative low affinity of the binding His-tag/metal ions (k_d_ = ~µM).

The authors intend to briefly explore the last strategy, which is the chosen one for the research presented in this paper. It involves utilizing a class of proteins known for their ability to bind antibodies. Protein A and protein G are derived from Staphylococcus aureus and Streptococcus aureus and by far the most used for this purpose. These proteins are known for possessing different domains specific for the Fc region of mammalian Igs. They have different binding proprieties and they are able to bind to distinct classes of Igs.

The binding is a non-covalent interaction, but there is no need for tagging the antibody of interest, which is a great advantage particularly when very limited amounts are available. The table (Table 1) reported below summarizes some of the advantages and disadvantages of the methods mentioned, adapted from Makaraviciute et al. [27].

The work presented in this research utilizes protein A-mediated binding for the oriented immobilization of IgG for the detection of fluorescent tubulin, confirming the data present in the literature. Protein A is covalently immobilized on the surface of gold clusters embedded in a silicon surface. The signal detection is conveyed through a metal enhanced fluorescence (MEF) system. Orienting immunoglobulins allows the exposition of the fragment antigen-binding (Fab) region for the binding to its antigen, substantially increasing the binding capacity per antibody immobilized.

## 2. Materials and Methods

### 2.1. Fabrication of Plasmonic Nanoparticles Clusters

P type (100) were used, and silicon wafers with a resistivity of 10 Ω/cm  were used as a substrate. The wafers were thoroughly cleaned with acetone, spin coated with the positive photo-resist S1813 (from Rohm and Haas, Philadelphia, PA, USA) at 4000 rpm for 60 s to achieve a thickness of 1 μm. Optical lithography (Karl Suss Mask Aligner MA 45, Suss MicroTec GA, Garching, Germany) was used to transfer in the resist a pattern of micro-holes with a size of d=10 μm and center-center distance of δ=30 μm. For the lithography, UV radiation with power density $P=2.5\;\mathrm{mW}/{\mathrm{cm}}^{2}$ was used for 30 s. After development in MF322 for 60 s, gold nanoparticles were deposited in the holes using site selective electroless deposition setting the process parameters as in reference (E. Battista et al., Metal enhanced fluorescence on super-hydrophobic clusters of gold nanoparticles. Microelectronic Engineering 175, 7–11, 2017). The residual resist was then removed from the sample surface with acetone, and the entire device was rinsed with DI water and dried under nitrogen flux.

### 2.2. Scanning Electron Microscopy (SEM) Images of Samples

Morphological characterization of nanoscale samples has been carried out by scanning electron microscopy (SEM). SEM images of the samples were captured using a Sigma 300VP (Zeiss, Germany) system. During the acquisitions, an accelerating voltage of 3 kV was used. More than 20 SEM images were acquired for each sample morphology.

### 2.3. Atomic Force Microscopy (AFM) Images of Samples

Samples of the SERS nano-islands were characterized by atomic force microscopy (combined Raman-AFM Witec alpha300 RA, Ulm, Germany). Images of 5 μm × 5 μm nano island areas were acquired in an intermittent, noncontact mode. Room temperature was held as fixed for all the acquisitions. Ultra-sharp silicon tips (FM-AC, 2.8 N/m Witec, Ulm, Germany) with a curvature radius at the tip less than 5 nm were used. The final images were averaged over multiple measurements, each of them performed at a scanning rate of 1 Hz. The images had a resolution of 256 × 256 points per line and were corrected using the Witec Project 2.10^®^ software. The characteristic power spectrum (PS) and then the fractal dimension were derived for all the substrates.

### 2.4. Functionalization of Gold Nanoparticles Clusters

The SERS substrates were sequentially functionalized by three different species: 1. mercaptoundecanoic acid (MUDA), 2. protein A from Staphylococcus aureus, both purchased from Merk, and 3. an antibody (Rabbit anti-tubulin, ABD Serotec or CD4 (VIT4) -FITC mouse anti human (Ab) from Miltenyi Biotec, in results section the antibody used in the specific experiment will be explicated). MUDA, [10 mM] in EtOH, was added to the AuNP islands, where it was spontaneously adsorbed through the thiol groups (-SH) which self-assembled on gold surface releasing hydrogen (A. Majzik et al. [58]). Furthermore, the chemistry of ethyl-3-[dimethylaminopropyl] carbodiimide hydrochloride (EDC) and N-hydroxy-succinimide (NHS) was employed to activate the surface, improving the covalent attachment of biomolecules relative to the next step. EDC (0.4 M) and NHS (0.1 M) solutions in PBS were mixed in 1:1 volume ratio, 25 μL was added dropwise and left to react on the chip for 7 min and then rinsed with PBS 1X. At this point two different samples were prepared: (a) chips without protein A, as control, and (b) chips with protein A and both were used to link antibodies. Total of 25 μL drop of protein A was deposited on the chip type b at a concentration of 0.2–1 mg/mL in PBS and left to react for 1 hr. After washing with PBS, a 25 μL drop of antibody (Ab) (Rabbit anti-tubulin, ABD Serotec) (1 mg/mL) was deposited on chips, both type a and b, and incubated for 1 h. Before analysis, chips were washed with PBS.

### 2.5. Raman Spectroscopy Analysis of Samples

Raman spectra of the gold nanoparticles after each step of functionalization and after the secondary antibody (antiAb) (goat anti-Rabbit IgG-HRP sc-2004 J3108. Santa Cruz Biotechnology. Concentration 200 μg/0.5 mL) trapping, were collected by a Renishaw InVia Microscope with a 1024 CCD detector, an excitation wavelength of 633 nm and a 50× objective lens. Mapping Raman measurements were carried out by a laser power of around 350 μW and a step size of 1 μm in the x- and y- axis directions. All map spectra were baseline-subtracted and analyzed using the free software package Raman Tool Set (freely available at http://ramantoolset.sourceforge.net accessed on 2 October 2019) [59]. Raman spectra of each single molecules in solution (as utilized during the sensing platform building) were collected by the drop deposition method, in the same measurement conditions of the functionalized AuNP, to constitute a small library for verifying the functionalization.

### 2.6. Acquiring Fluorescence Images of Samples

Solutions of FITC fluorescent molecules (antibodies or antigens as specified in Results section) were deposited on the chips by the drop method and left to react for 1 h. Washed samples were analyzed using Nikon, ECLIPSE Ti fluorescent microscope (Nikon Instruments Inc., Melville, NY, USA), with an excitation length of λex = 488 nm, which gives the functionalized gold islands green fluorescence.

### 2.7. Fluorescence Testing on Antibody Immobilization

Antibody CD4 (VIT4)—FTIC mouse snit-human, Miltenyi Biotec, was immobilized on substrate with and without protA to compare the immobilization ability of the two kinds of configurations. The solutions used for functionalization were analyzed after the immobilization process by a VICTOR3 Multilabel Plate Reader (PerkinElmer, Inc., Waltham, MA, USA), which quantify the fluorescence intensity (FI) in solution. A calibration curve was realized to quantify the antibody remained in solution. At the same time, the devices with the immobilized antibody were analyzed by the fluorescent microscopy.

## 3. Results

### 3.1. Clusters of Plasmonic Gold Nanoparticles

**Figure 3:**
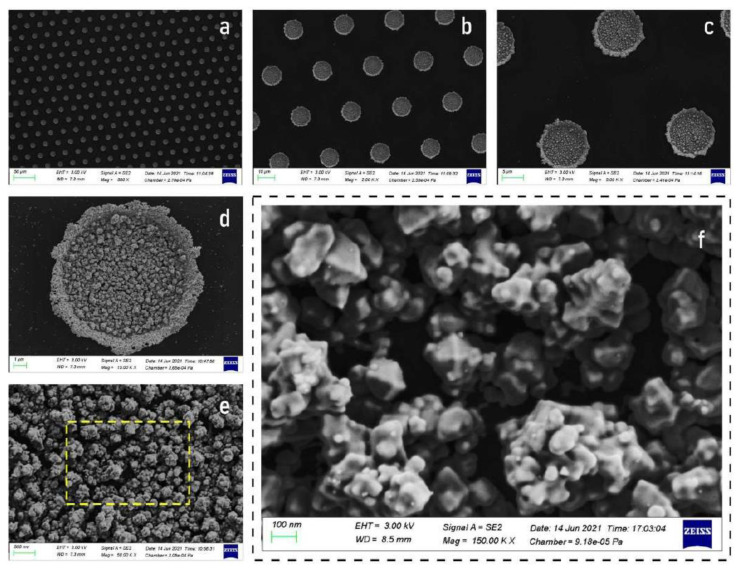
SEM images of gold nanoparticles clusters taken at low magnification reveal the fabrication process capability to attain maximum control over the shape and the size of the clusters (**a**–**c**). SEM micrographs of the samples taken at higher magnification (**d**–**f**) illustrate the particle morphology at the nanoscale.

Using the procedures described in the Methods of the paper, clusters of gold nanoparticle on substrates of silicon were fabricated. Scanning electron microscopy (SEM) micrographs of the samples were taken to assess repeatability and reproducibility of the fabrication method. SEM images with low magnification factor from 500× to 5000× (Figure 3a–c) illustrate that the clusters of gold nanoparticles extend over areas of several hundred μm with no interstitial or vacancy defects in the patterns that are predominantly regular in this dimensional interval. The clusters of gold nanoparticles have a diameter of approximately 10 μm and are deposited in a lattice with hexagonal crystalline structure and spacing between elements of 20 μm. SEM images of the samples taken at higher magnification (Figure 3d,e) show that, in each cluster, gold nanoparticles are densely packed, exhibiting an imperfect rhombohedric shape and a size that ranges from about 50 nm to nearly 200 nm for the larger structures (Figure 3f). The extent of this scale interval suggests that particles in the cluster may have a dendritic structure, with smaller particles more numerous than larger particles and the same geometrical motifs occurring periodically at different length scales.

### 3.2. Atomic Force Microscopy

**Figure 4:**
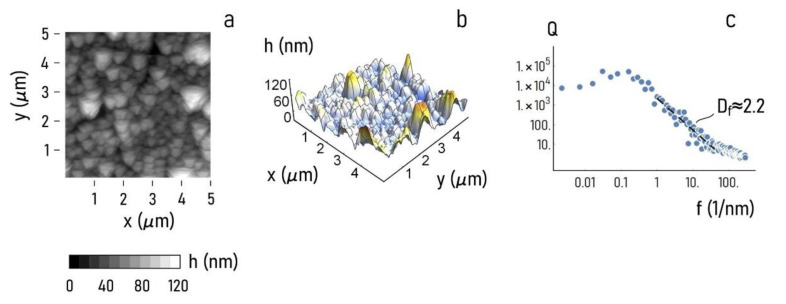
Atomic force microscopy (AFM) was used to image the gold nanoparticles with maximum resolution. Surface density plot (**a**) and 3D (**b**) plots of the AFM surface profile show that the maximum particle height in a 5 × 5 μm^2^ scan area is about 120 nm^2^. Power spectrum density function extracted from the AFM scan reveals the hierarchical structure of the sample surface at the nanoscale (**c**).

To verify this hypothesis (Methods), atomic force microscopy (AFM) images of the sample surface were acquired (Figure 4). The relief density plot and the 3D plot of the AFM scan are reported in Figure 4a,b. AFM images show that the height profile of the particles falls in the 0−60 nm interval, with fewer sample points reaching the 80−120 nm limit. We found the power spectrum density (Q) associated to these profiles using the methods reported by F. Gentile et al. [60]. The power spectrum density illustrates how the information content of an image changes as a function of scale. Then, Q was used to calculate the fractal dimension of the sample surface as $D_{f}\sim 2.2$ (Figure 4c). The fractal dimension is a non-dimensional number comprising between 2 and 3 that indicates how much of the surface detail is conserved across different scales. The value of fractal dimension of $D_{f}\sim 2.2$ that we have found for this sample surface indicates that the clusters of gold nanoparticles have a complexity higher than that associated to a Euclidean surface, with = 2. 

SERS (surface enhanced Raman scattering) and MEF (metal enhanced fluorescence) effects are largely influenced by particle shape and size, and by the constituting material of the nanostructured surface. As regarding particle size, a number of studies reported in literature have illustrated that SERS and MEF devices perform efficiently in the 10–140 nm dimensional range, and reach an optimum for a size of approximately 50 to 60 nm [61,62,63,64,65]. Notably, the gold nanostructures that we have used for this study have a size that falls in this interval. While this work was not focused on optimizing performance, and was more an exercise on existence of proof, in a more sophisticated evolution of the device that will be developed over time we will harness the nanofabrication techniques to produce nanoparticles with a tight control over their shape and size. This will enable us to optimize the enhancement of the sensor device.

### 3.3. Fluorescence Analysis for MEF Effect Evaluation

**Figure 5:**
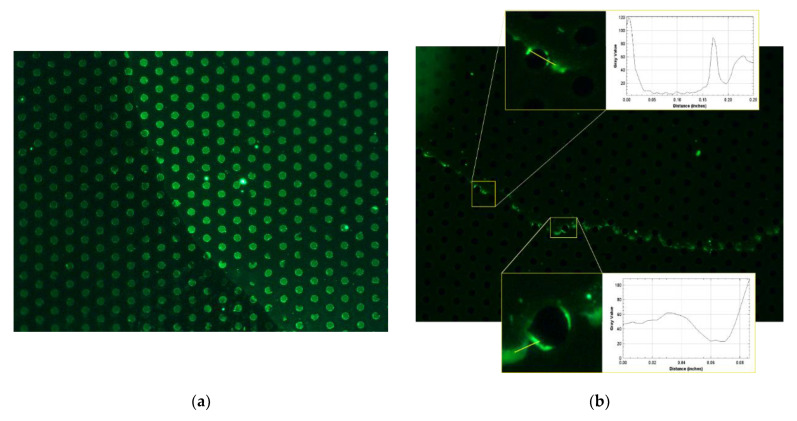
Fluorescent image of washed a Ab FITC drop [1 µg/mL] (**a**) and the lowest concentration recorded [100 fg/mL] (**b**). The zoom areas around the AuNPs islands highlight the MEF effect, evidenced by the intensity value of fluorescence, calculated along the yellow line plot in each zoom.

Drops at different dilutions (2 mg/mL ÷ 100 fg/mL) of antibody Ab FITC were deposited on the AuNP array platform and observed, after drop drying, under the fluorescent microscope. The lower visible concentration was around 100 fg/mL. Fluorescence is significative on the coffee ring generated by the drop drying (Figure 5b), furthermore in this area the enhancement ability of the gold nanograins (AuNPs) is evident, in particular on the AuNPs islands’ edges. Intensity of the fluorescent signal is increased by an average of around 48%. In Figure 5 two representative areas around AuNPs islands were zoomed: the MEF effect is particularly present on the edges of the metallic islands the intensity value of fluorescence was calculated along the yellow line plot in each zoom area.

In addition, 1 µg/mL of FITC-Ab was used to verify the influence of the metal enhanced fluorescence effect (MEF) given by the AuNPs. As shown in Figure 5a, the fluorescence recorded in the AuNPs is significantly higher than the one recorded at the same concentration in the absence of AuNPs resulting in a up to 500% increase in signal recorded.

### 3.4. Characterization of the Functionalized Plasmonic Gold Nanoparticles

**Figure 6:**
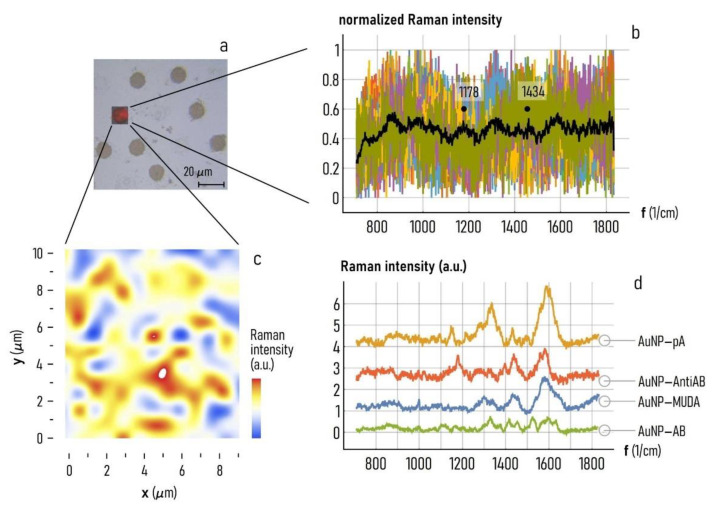
(**a**) Optical image of AuNP array template, analyzed by Raman spectroscopy. (**b**) Raman spectra collected during a mapping analysis. The averaged spectrum (black) evidence the two significative peaks at points 1178 cm^−1^ (the histidine and tyrosine signal) and 1434 cm^−1^ (CH_2_ bending peak). (**c**) Map of distribution of the Raman intensity for the 1178 cm^−1^ peak. (**d**) Raman spectra of the different functionalization steps.

Raman spectroscopy on AuNPs’ islands allowed to evaluate the AntiAB entrapping ability. The collected Raman maps were analyzed revealing intensity at points 1178 cm^−1^ and 1434 cm^−1^ (Figure 6a,b), respectively the histidine and tyrosine signals and the CH_2_ bending peak [66].

Figure 6c shows the distribution of the Raman intensity for the 1178 cm^−1^ peak, but the same results were obtained for the 1434 cm^−1^ point (data not reported), evidencing a homogeneous distribution of the secondary antibody on the AuNPs, while no signal was from the surrounding area.

How the functionalization occurs, step by step, until the building of the final arrayed sensing platform was also followed by the Raman analyses. As detailed in Methods, the SERS active-regions of AuNPs’ islands were first activated by a SAM (self-assembled monolayer) technique, when MUDA molecules are spontaneously adsorbed on gold by the SH groups. The other terminal group of MUDA is a COOH- moiety which, helped by EDC/NHS complex, works as anchor to link protein-type molecules. Protein A engages the carboxylic group promoting the following antibodies binding in the correct orientation.

Raman spectra of the different functionalization steps (Figure 6d) evidence an initially increments of the typical proteins’ signals in the passage between MUDA and protein A functionalization: the peak of amide I around 1630 cm^−1^ is present as a shoulder assigned to the C=O stretching of the carbonyl groups, also presents in the MUDA molecules, nevertheless the peak at ~1335 cm^−1^, attributable to NH bending and C–N stretching mode of amide III, is proportionally increased with respect to the total spectrum after protein A functionalization as well as the backbone skeletal stretch signal at ~1170 cm^−1^ [67].

The antibody-protein A interaction does not give a prominent difference in Raman fingerprint. What it is observable, instead, is a global decrease or the Raman intensity, due to the incremented distance between the added molecules and the SERS substrate [68].

Moreover, tryptophan in proteins, peak at ~1550 cm^−1^, is one of the amino acid more influenced by proteins’ aggregation or conformational changes due to external stimuli. The linkage between protein A and the antibody is precisely highlighted by the increment of the 1550 cm^−1^ peak, recognizing changes in both secondary and tertiary structure of the antibody [67].

When the interaction with the secondary antibody occurs, the total signal continues to decrease and only the, already known, 1434 cm^−1^ (CH2 bending) peak and the histidine and tyrosine signal at 1178 cm^−1^ become more prominent [66].

**Figure 7:**
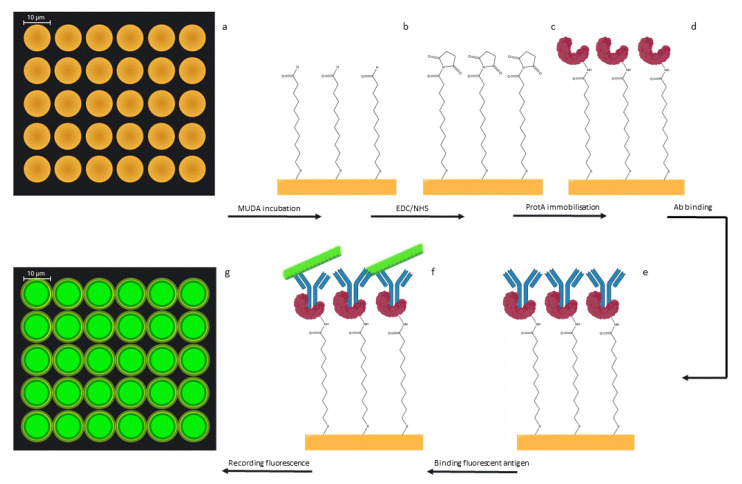
Schematic representation of the silicon platform functionalization. Silicon surface with gold clusters (**a**), overnight incubation with MUDA (**b**), EDC/NHS activation (**c**), protein A covalent immobilization (**d**), protein A binding anti-tubulin Ab (**e**), anti-tubulin Ab binding fluorescent tubulin (**f**), schematic representation of fluorescence recorded (**g**). Image created with BioRender.com (accessed on 7 August 2021).

After overnight incubation in MUDA, protein A (protA) was covalently immobilized on the functionalized gold clusters through standard EDC/NHS covalent immobilization (Figure 7a–d). Although many techniques are known in the literature for the functionalization of gold surfaces, such as the use of dry NHS [69] or through avidin conjugation [70], in this work, MUDA-mediated functionalization was used to allow COOH groups to be deposited on the gold surface and subsequently immobilize proteins through EDC/NHS chemistry; established immobilization procedure for biological elements [71] Protein A is known for binding IgG through its Fc region, it was therefore used by the authors to vehicle the oriented immobilization of IgG.

In order to verify the oriented binding, an anti-tubulin binding antigen was used as model antibody (Ab) and fluorescent tubulin (antigen) was used for visualizing the correct immobilization. If IgG is correctly oriented on the surface through the binding with protein A, it would expose the Fab region and make it accessible for the binding to its antigen (tubulin), effectively increasing its binding capacity.

ProtA functionalized surface was incubated with anti-tubulin to allow the binding, and after PBS washes, fluorescent tubulin was added, followed by final washes to remove the excess of un-bound tubulin. Fluorescence was recorded (Figure 7e–g).

To assess the orientation, the experiment was conducted in parallel to a surface functionalized with anti-tubulin Ab covalently immobilized on the gold clusters in the absence of protein A, followed by the addition of fluorescent tubulin.

**Figure 8:**
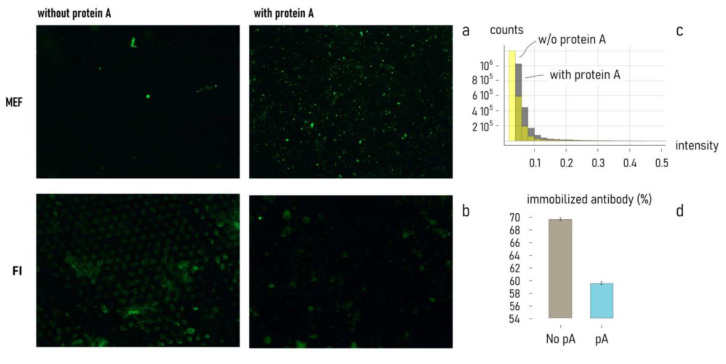
Fluorescence recorded without protA, and therefore with anti-tubulin randomly immobilized binding fluorescent tubulin (**a** left), compared to the surface with protA orienting anti-tubulin binding (**a** right). The images recorded show an increase in fluorescence recorded of 59% in the surface with the presence of protA. Fluorescent IgG randomly immobilized (**b** left) compared to fluorescent IgG bound by protA, higher fluorescence recorded when IgG is randomly immobilized, resulting in higher concentration immobilized. Graph (**c**) show the counts of fluorescent points in relation to their intensity for the **a** line images. The percentage of immobilized IgG is showed on Graph (**d**) where the immobilized antibody was calculated by an FI test on the solution used for antibody immobilization.

Figure 8 shows the platform with fluorescent recorded in the presence of protein A compared to the platform with the same concentration anti-tubulin covalently and randomly immobilized on the surface (Figure 8). Based on the recorded fluorescence, it is observable how the surface where the binding is vehicled by protA there is an increment of fluorescent recorded of 59 percentage, indicating a better exposure of the antibody fab region to its antigen.

In addition, the amount of antibody randomly immobilized and bound to protA was quantified using a fluorescent antibody as model in fluorescence intensity (FI) assay.

Figure 8, FI line, shows how even though the same concentration of antibody was originally used in the functionalization, covalently immobilizing it allows the immobilization of a greater amount of it (higher fluorescence recorded). Nevertheless, the amount of tubulin bound, results in higher presence of protein A. The two results combined show how even with higher concentration of Ab immobilized, the orientation is the most important factor to consider.

## 4. Discussion

In this article, we present a specific method to obtain a functionalized surface for immunodetection to overcome challenges of low-abundance biomarker and for improving signal to noise for a visible detection that is more successful [72].

The outcome in the use of protein A for a correct spatial disposition of the antibody delegated to target detection is not only improving the performance of the assay, but also reducing the abundance of antibody solution to sacrifice. This result is explained by the role of protein A. In fact, its binding with the tail (Fc region) of the antibody makes all its ligand-sites (Fab region) spatially available to bind the target. On the contrary, the absence of protein A determines a random disposition of the antibodies on the surface. This no orientated disposition of antibody resulted in a performance of linking of around 70% from the starting solution used in the functionalization. When protein A is previously linked on the surface, to functionalize the same area, less than 60% of the starting solution was used for the immobilization of the antibody. Moreover, the detection power is amplified by the right orientation of the all-ligand sites and, adding the enhancing effect of the metallic nanostructures, a total increment of fluorescence of 59% is reached with respect to the same area randomly functionalized. This molecular construct, combined with the MEF structures achieves a strong signal intensity and conveys high-quality detection, for the consistency of the data and providing performance equal to or better than traditional analytical approaches as immunoassays ELISAs-like (enzyme-linked immunosorbent assay) and IHC (Immunohistochemistry) [73,74].

Several different formats are used in ELISAs, but they all fall into either direct, indirect or sandwich capture and detection methods. The antigen is then detected either directly (labeled primary antibody) or indirectly (such as labeled secondary antibody). The most used ELISA format is sandwich ELISA assay, where the specific antibody (capturing antibody) is immobilized on the surface of the plate to bind the target analyte and either an antibody directly binding the analyte (primary antibody) is tagged with an enzyme producing colorimetric signal or a fluorescent tag. Alternatively, to the specific primary untagged antibody a tagged antibody binding the primary antibody (secondary antibody) is added, generating the signal. Due to the high versatility and the lack of need for tagging the primary antibody, this last type is the most commonly used assay in diagnostics) [73,74].

In surface presented in this research, target detection is competitive with both traditional methods, as it demonstrates a good target detection performance even at low concentrations, competitive with ELISA assays, and at the same time qualifies the detection through imaging given by the fluorescent signal, similarly to qualitative colorimetric data resulting by applying IHC assay. Furthermore, the fluorescent signal generated by the surface represents an intrinsic test of verification and validation of the specificity of binding between target and antibody, suggesting also the widest dynamic range of functionalized gold nanoparticles necessary to detect it [75].

Adding a deep learning machine to a device built with this protocol highlights the limitations of the traditional marker detection system, opening up the delivery of low abundance markers, and making them available in complex matrices for combined quantitative and qualitative evaluation [76].

## 5. Conclusions

Separation by immunoaffinity and diagnostics are the two main fields of application of antibodies [77].

The main component in clinical disease diagnostics are antibody-based immunoassays. Most of the point-of-care used in personalized monitoring of chronic degenerative diseases is mainly based on the use of antibodies directed against the biomarker of interest. Moreover, the fabrication and demand for specific point-of-care for specific diseases with epidemiological impact, is growing rapidly in the international Healthcare market [78].

For this reason, identifying of low abundance biomarkers in complex matrices by combining quantitative data with qualitative ones is now an imperative requirement. In addition, peripheral blood or other body fluids sampled in a non-invasive way are the most investigated biological matrices. The surface presented here, fulfils the two requirements, quantization of low target concentrations and qualitative demonstration of its detection. With these assumptions, the surface created here represents the basis for making lab-on-chips with a high degree of operational performance, robustness, specificity, and sensitivity useful for visible discrimination of the target in any type of complex matrices.

## Figures and Tables

**Table 1:** Comparison of different site-directed and random antibody immobilization techniques, table adapted from Makaraviciute et al. Reprinted with permission from Ref. [27]. Copyright 2013 Elsevier.

Immobilization Format	Methodology	Advantages	Disadvantages
Random	Covalent attachment via amine coupling Physiosorption	In some cases shows good sensitivity [28,29] Surface regeneration for multiple analyses after covalent attachment [30]	Lower sensitivity in comparison to site-directed immobilization methods [31] In case of physiosorption denaturation of proteins, very low stability and random protein orientation [32,33]
Via His-tag	Expression of recombinant antibody with His-tag	- Oriented binding through His-tag/metal ions interaction	- Labelling of the antibody required - Low affinity (K_d_ = 10^−6^ M) - Competition with metal endogenous proteins [34]
Via biotinylated antibody	Site specific biotinylation	- Oriented binding through the strongest known non-covalent interaction (K_d_ = 10^−15^ M) [23,35] - Rapid bond, not affected by extremes of pH, temperature, or organic solvents [35]	- Labelling of the antibodies required - High background signal due to the presence of endogenous biotin in tissues [36] - Need for blocking endogenous biotin
Via an oxidized oligosaccharide moiety	Chemical or enzymatic oxidation of an oligosaccharide moiety and coupling to amine or hydrazine terminated supports	- Improvement in sensitivity in comparison to random immobilization [30,37] - No direct modification of amino acids [37,38] - Surface regeneration for multiple analyses [30]	- Different reactions conditions, such as temperature, pH, and periodate concentration, might strongly affect oxidation and can yield inconsistent results [39] - Antibody structure damage during oxidation especially for certain mABs [40]
Via antibody fragments	Chemical reduction or genetic engineering based disruption of disulfide bridges and immobilization via sulfhydryl groups	- Improvement in sensitivity in comparison to random immobilization [41,42] - Affinity towards antigen is adjustable of recombinant Fab’ [43] - Surface regeneration for multiple analyses [44]	- Steric hindrance possibility because of a very compact layer [45,46] - Chemical reduction resulting in the potential loss of biological activity of Ab fragments, especially in the case of mAbs (monoclonal antibodies) [47,48,49] - Low stability of genetically engineered fragments [50] - Possible Ab denaturation upon direct contact with gold [51] - Which is likely to cause non- specific binding [52]
Site-directed Via Fc binding proteins	Affinity interactions with a preformed layer of proteins specific to the Fc regions of Ab, e.g., proteins A, G, A/G, L, anti-Fc, recombinant proteins	- Improvement in sensitivity in comparison to random immobilization [31,53] - Does not require antibody modification [54,55] - Surface regeneration for multiple analyses if cross-linking is used [30,45]	- Single use if non-cross linked [30,44] - Cross-linking might reduce sensitivity [53,56] - Protein G is reported to be prone to non-specific interactions [57] - Specific to certain classes of antibodies only [57]

## Data Availability

Not applicable.
